# Role of Plastid Protein Phosphatase TAP38 in LHCII Dephosphorylation and Thylakoid Electron Flow

**DOI:** 10.1371/journal.pbio.1000288

**Published:** 2010-01-26

**Authors:** Mathias Pribil, Paolo Pesaresi, Alexander Hertle, Roberto Barbato, Dario Leister

**Affiliations:** 1 Plant Molecular Biology (Botany), Department Biology I, Ludwig-Maximilians-Universität, Munich, Germany; 2 Mass Spectrometry Unit, Department Biology I, Ludwig-Maximilians-Universität, Munich, Germany; 3 Department of Biomolecular Sciences and Biotechnology, University of Milan, Milan, Italy; 4 Department of Environmental and Life Sciences, Università del Piemonte Orientale, Alessandria, Italy; The Salk Institute for Biological Studies, United States of America

## Abstract

Short-term changes in illumination elicit alterations in thylakoid protein phosphorylation and reorganization of the photosynthetic machinery. Phosphorylation of LHCII, the light-harvesting complex of photosystem II, facilitates its relocation to photosystem I and permits excitation energy redistribution between the photosystems (state transitions). The protein kinase STN7 is required for LHCII phosphorylation and state transitions in the flowering plant *Arabidopsis thaliana*. LHCII phosphorylation is reversible, but extensive efforts to identify the protein phosphatase(s) that dephosphorylate LHCII have been unsuccessful. Here, we show that the thylakoid-associated phosphatase TAP38 is required for LHCII dephosphorylation and for the transition from state 2 to state 1 in *A. thaliana*. In *tap38* mutants, thylakoid electron flow is enhanced, resulting in more rapid growth under constant low-light regimes. *TAP38* gene overexpression markedly decreases LHCII phosphorylation and inhibits state 1→2 transition, thus mimicking the *stn7* phenotype. Furthermore, the recombinant TAP38 protein is able, in an in vitro assay, to directly dephosphorylate LHCII. The dependence of LHCII dephosphorylation upon TAP38 dosage, together with the in vitro TAP38-mediated dephosphorylation of LHCII, suggests that TAP38 directly acts on LHCII. Although reversible phosphorylation of LHCII and state transitions are crucial for plant fitness under natural light conditions, LHCII hyperphosphorylation associated with an arrest of photosynthesis in state 2 due to inactivation of TAP38 improves photosynthetic performance and plant growth under state 2-favoring light conditions.

## Abstract 2

Regulation of photosynthesis efficiency involves reversible phosphorylation of the light-harvesting complex through the activity of the newly identified phosphatase TAP38.

## Author Summary

Plants are able to adapt photosynthesis to changes in light levels by adjusting the activities of their two photosystems, the structures responsible for light energy capture. During a process called *state transitions*, a part of the photosynthetic complex responsible for light harvesting (the photosynthetic antennae) becomes reversibly phosphorylated and migrates between the photosystems to redistribute light-derived energy. The protein kinase responsible for phosphorylating photosynthetic antenna proteins was identified recently. However, despite extensive biochemical efforts to isolate the enzyme that catalyzes the corresponding dephosphorylation reaction, the identity of this protein phosphatase has remained unknown. In this study, we identified and characterized the thylakoid-associated phosphatase TAP38. We first demonstrate by spectroscopic measurements that the redistribution of excitation energy between photosystems that are characteristic of state transitions do not take place in plants without a functional TAP38 protein. We then show that the phosphorylation of photosynthetic antenna proteins is markedly increased in plants without TAP38, but decreased in plants that express more TAP38 protein than wild-type plants. This, together with the observation that addition of recombinant TAP38 decreases the level of antenna protein phosphorylation in an in vitro assay, suggests that TAP38 directly acts on the photosynthetic antenna proteins as the critical phosphatase regulating state transitions. Moreover, in plants without TAP38, photosynthetic electron flow is enhanced, resulting in more rapid growth under constant low-light regimes, thus providing the first instance of a mutant plant with improved photosynthesis.

## Introduction

Owing to their sessile life style, plants have to cope with environmental changes in their habitats, such as fluctuations in the incident light. Changes in light quantity or quality (i.e., spectral composition) result in imbalanced excitation of the two photosystems and decrease the efficiency of the photosynthetic light reactions. Plants can counteract such excitation imbalances within minutes by a mechanism called *state transitions*, which depends on the reversible association of the mobile pool of major light-harvesting (LHCII) proteins with photosystem II (state 1) or photosystem I (PSI) (state 2) (reviewed in [1]–[5]). In detail, the accumulation of phosphorylated LHCII (pLHCII), stimulated in low white light, or by light of wavelengths specifically exciting PSII (red light), causes association of pLHCII with PSI (state 2), thus directing additional excitation energy to PSI. Conditions like darkness or light of wavelengths specifically exciting PSI (far-red light), as well as high intensities of white light, stimulate pLHCII dephosphorylation and its migration to PSII (state 1), thus redirecting excitation energy to PSII.

LHCII phosphorylation and state transitions have been extensively studied in the green alga *Chlamydomonas reinhardtii* and the flowering plant *Arabidopsis thaliana* [2],[4]–[6]. In *C. reinhardtii*, the impact of state transitions on interphotosystem energy balancing and on promoting cyclic electron flow is well established [2],[5]. In flowering plants, however, the physiological significance of state transitions is less clear, because their mobile LHCII pools are significantly smaller than those in green algae [7],[8]. Thus, *A. thaliana* mutant plants impaired in state transitions are only marginally affected in their development and fitness [9]–[11], even under fluctuating light or field conditions [12],[13]. However, when *Arabidopsis* state transition mutants are perturbed in linear electron flow, effects on plant performance and growth rate become evident [14], indicating that also in flowering plants, state transitions are physiologically relevant.

The protein kinase responsible for phosphorylating LHCII is membrane bound and activated upon reduction of the cytochrome *b*_6_/*f* (Cyt *b*_6_/*f*) complex via the plastoquinone (PQ) pool under state 2-promoting light conditions (low white light or red light) [15],[16]. PQ oxidizing conditions induced by state 1-promoting light conditions (dark or far-red light) inactivate the LHCII kinase and result in association of pLHCII with PSII (state 1, reviewed in [4],[5]). The LHCII kinase activity, however, is also inactivated under high white light conditions, when the stromal reduction state is very high. In vitro and, more recently, in vivo studies suggest that suppression of LHCII kinase activity might be mediated by reduced thioredoxin [17],[18]. In *C. reinhardtii* and *A. thaliana*, the orthologous thylakoid protein kinases Stt7 and STN7, respectively, are required for LHCII phosphorylation and state transitions [12],[19]. Coimmunoprecipitation assays showed that the Stt7 kinase interacts with Cyt *b*_6_/*f*, PSI, and LHCII [17], suggesting that Stt7 (and STN7 in *Arabidopsis*) directly phosphorylates LHCII, rather than being part of a Stt7/STN7-dependent phosphorylation cascade.

Under PQ oxidizing conditions when the LHCII kinase becomes inactivated, pLHCII is dephosphorylated by the action of an as-yet unknown protein phosphatase, thus allowing the association of the mobile fraction of LHCII with PSII (state 1) [5],[7]. For many years, attempts were undertaken to elucidate the characteristics and to identify the LHCII protein phosphatase(s). By means of biochemical approaches [20]–[22], it was shown that protein phosphatases of different families must be involved in the reversible phosphorylation of thylakoid phosphoproteins. A PP2A-like phosphatase was postulated to be responsible for the desphosphorylation of the PSII core proteins [23], whereas the LHCII phosphatase activity was shown to be dependent on the presence of divalent cations and not to be inhibited by microcystin and okadaic acid [21],[22]. These findings strongly suggested an involvement of a PP2C-type phosphatase in pLHCII dephosphorylation [24].

Here, we show that the thylakoid protein phosphatase TAP38 is required for pLHCII dephosphorylation and state transitions. In plants with markedly reduced TAP38 levels, hyperphosphorylation of LHCII is associated with enhanced thylakoid electron flow, resulting in more rapid growth under constant low-light regimes. Together with the results of an in vitro dephosphorylation assay, our data indicate that TAP38 dephosphorylates pLHCII directly.

## Results

### Screening for Candidate LHCII Phosphatases

To identify the LHCII phosphatase, we systematically isolated loss-of-function mutants for known chloroplast protein phosphatases and assessed their capacity to dephosphorylate pLHCII (see below for details). However, none of the nine protein phosphatases At3g52180 (DSP4/SEX4), At4g21210 (AtRP1), At1g07160, At3g30020, At4g33500, At1g67820, At2g30170, At3g10940, or At4g03415, demonstrated to reside in the chloroplast [25]–[29], qualified as the LHCII phosphatase (unpublished data). Next, we extended our search to protein phosphatases tentatively identified as chloroplast proteins by proteomic analyses in *A. thaliana* [30],[31]. Of those, the serine/threonine protein phosphatase At4g27800 turned out to be the most promising candidate.

### At4g27800.1 (TAP38) Is a Thylakoid-Associated Protein Phosphatase

**Figure 1:**
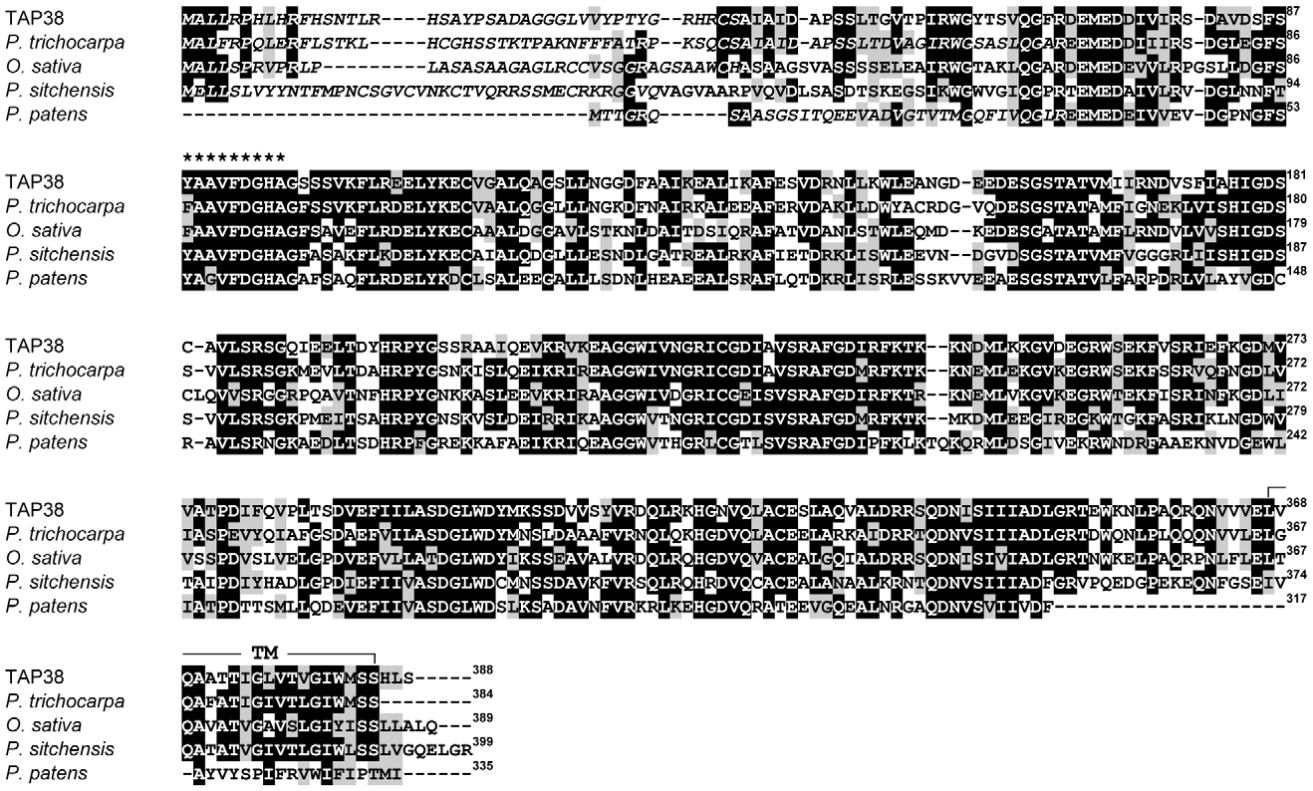
Comparison of the TAP38 sequence with those of related proteins from higher plants and moss. The amino acid sequence of the *Arabidopsis* TAP38 protein (At4g27800) was compared with related sequences from *Populus trichocarpa* (POPTRDRAFT_250893), *Oryza sativa* (Os01g0552300), *Picea sitchensis* (GenBank: EF676359.1), and *Physcomitrella patens* (PHYPADRAFT_113608). Black boxes highlight strictly conserved amino acids, and gray boxes closely related ones. Amino acids that constitute the protein phosphatase 2C signature are indicated by asterisks. Putative chloroplast transit peptides (cTPs) are indicated in italics, and the potential transmembrane domain (TM) is highlighted.

Proteins with high homology to At4g27800 exist in mosses and higher plants, but not in algae or prokaryotes. Furthermore, At4g27800 and its homologs share a predicted N-terminal chloroplast transit peptide (cTP), a putative transmembrane domain at their very C-terminus and a protein phosphatase 2C signature (Figure 1). For *A. thaliana*, three *At4g27800* mRNAs are predicted (Figure S1A). To verify their existence and to distinguish between the different splice forms, reverse-transcriptase PCR analyses were performed. Only *At4g27800.1*, and much less *At4g27800.2*, were detectable in leaves, whereas for the *At4g27800.3* splice variant, no signal could be obtained (Figure S1B).

**Figure 2:**
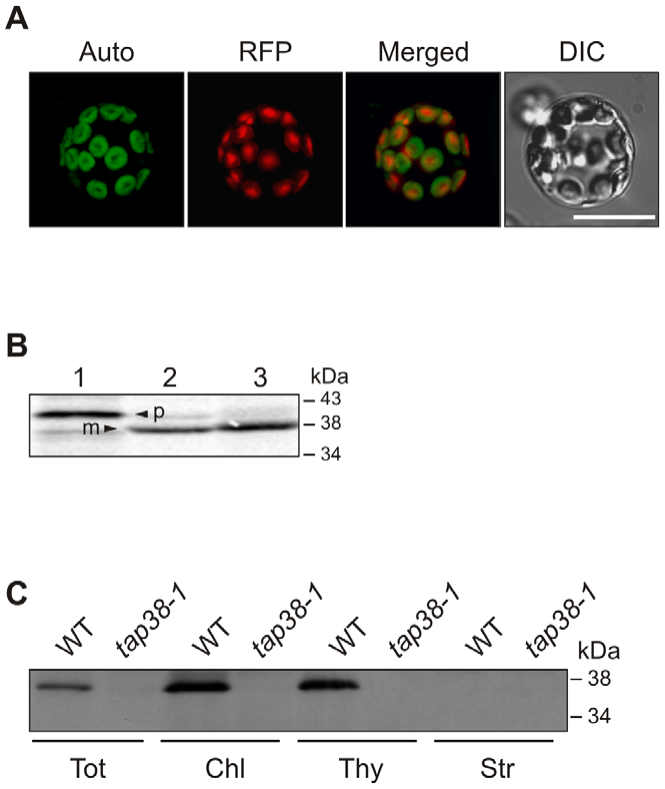
Subcellular localization of TAP38. (A) Full-length TAP38-RFP was transiently expressed in *Arabidopsis* protoplasts and visualized by fluorescence microscopy. Auto, chlorophyll autofluorescence; DIC, differential interference contrast image; merged, overlay of the two signals; RFP, fusion protein. Scale bar indicates 50 µm. (B) ^35^S-labeled TAP38 protein, translated in vitro (lane 1, 10% translation product), was incubated with isolated chloroplasts (lane 2), which were subsequently treated with thermolysin to remove adhering precursor proteins (lane 3), prior to SDS-PAGE and autoradiography. m, mature protein; p, precursor. (C) Immunoblot analyses of proteins from WT and *tap38-1* leaves. Equal protein amounts were loaded. Filters were immunolabeled with a TAP38-specific antibody. Chl, total chloroplasts; Str, stromal proteins; Thy, thylakoid proteins; Tot, total protein.

In protoplasts transfected with *At4g27800.1* fused to the coding sequence for the red fluorescent protein (RFP) [32], the fusion protein localized to chloroplasts (Figure 2A). Chloroplast import assays with the radioactively labeled At4g27800.1 protein confirmed the uptake into the chloroplast with concomitant removal of its cTP. Mature At4g27800.1 has a molecular weight of ∼38 kDa (Figure 2B). Immunoblot analysis using a specific antibody raised against the mature At4g27800.1 protein (Figure 2C) detected the protein in thylakoid preparations but not in stromal fractions. It is noteworthy, that the putative translation products At4g27800.2 and At4g27800.3 (∼32 kDa) were undetectable (Figure 2C). At4g27800.1 is therefore the major isoform in leaves, and was renamed TAP38 (Thylakoid-Associated Phosphatase of 38 kDa).

### TAP38 Expression in Wild-Type, *tap38* Mutant, and TAP38 Overexpressor Plants

**Figure 3:**
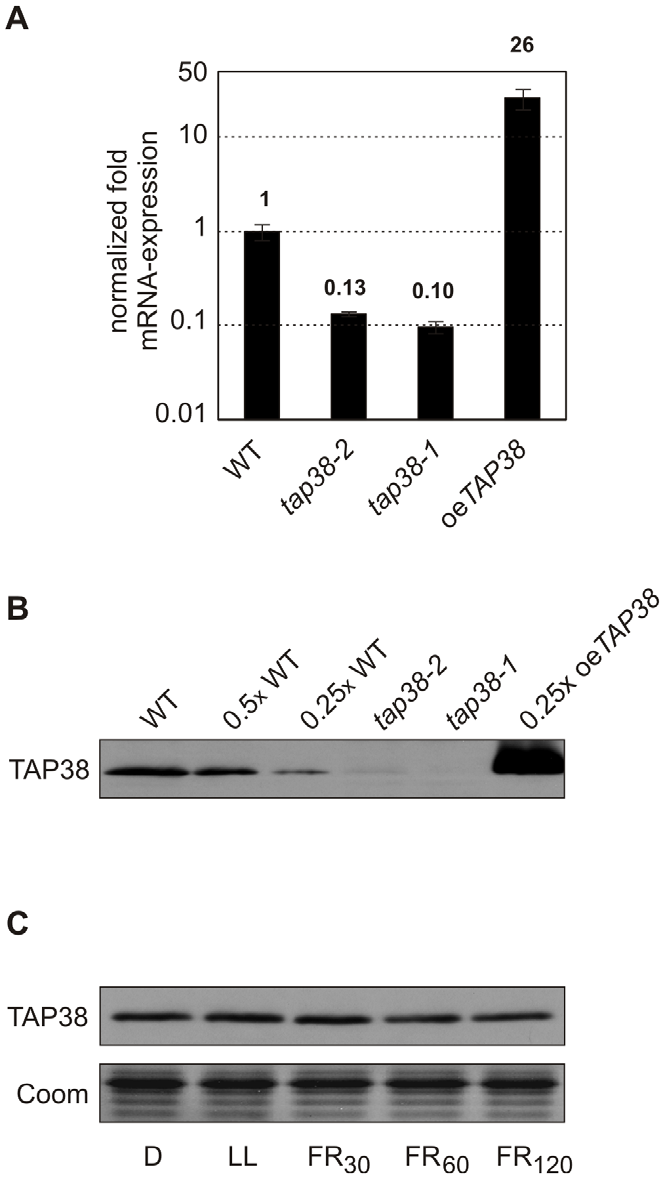
Expression of TAP38 in *tap38* mutant, TAP38 overexpressor, and WT plants. (A) Quantification of *TAP38* mRNAs by real-time PCR in WT, *tap38-1*, *tap38-2*, and oe*TAP38* leaves using the primer combination 1 and 2 (as in Figure S1A and S1B). (B) Thylakoid proteins from WT and *tap38* mutants were loaded in the corresponding lanes. Reduced amounts of oe*TAP38* thylakoids, corresponding to 25% of WT amount were loaded in the lane marked as 0.25× oe*TAP38*. Additionally, decreasing levels of WT thylakoids were loaded in the lanes indicated as 0.5× WT and 0.25× WT. Filters were immunolabeled with a TAP38-specific antibody raised against the mature TAP38 protein. (C) Thylakoid membranes of WT plants exposed to different light conditions (see Figure 5) were separated by SDS-PAGE. Immunodecoration of the corresponding Western blot was performed using a TAP38-specific antibody raised against the mature protein. A detail of a replicate gel, corresponding to the LHCII migration region, stained with Coomassie Blue is shown as loading control.

Two *tap38* insertion mutants, *tap38-1* (SAIL_514_C03) [33] and *tap38-2* (SALK_025713) [34], were obtained from T-DNA insertion collections (Figure S1A). In *tap38-1* and *tap38-2* plants, amounts of *TAP38* transcripts were severely reduced, to 10% and 13% of WT levels, respectively (Figure 3A). Conversely, in transgenic lines carrying the *TAP38* coding sequence under control of the 35S promoter of Cauliflower Mosaic Virus (oe*TAP38*), levels of *TAP38* mRNA were much higher than in wild type (WT) (Figure 3A). TAP38 protein concentrations reflected the abundance of *TAP38* transcripts: *tap38-1* and *tap38-2* thylakoids had <5% and ∼10% of WT levels, respectively, whereas the oe*TAP38* plants displayed >20-fold overexpression on the protein level (Figure 3B). TAP38 protein levels were also determined under light conditions relevant for state transitions (see Materials and Methods). In WT plants, TAP38 was constitutively expressed at similar levels under all light conditions applied (Figure 3C).

### TAP38 Is Required for State Transitions

**Figure 4:**
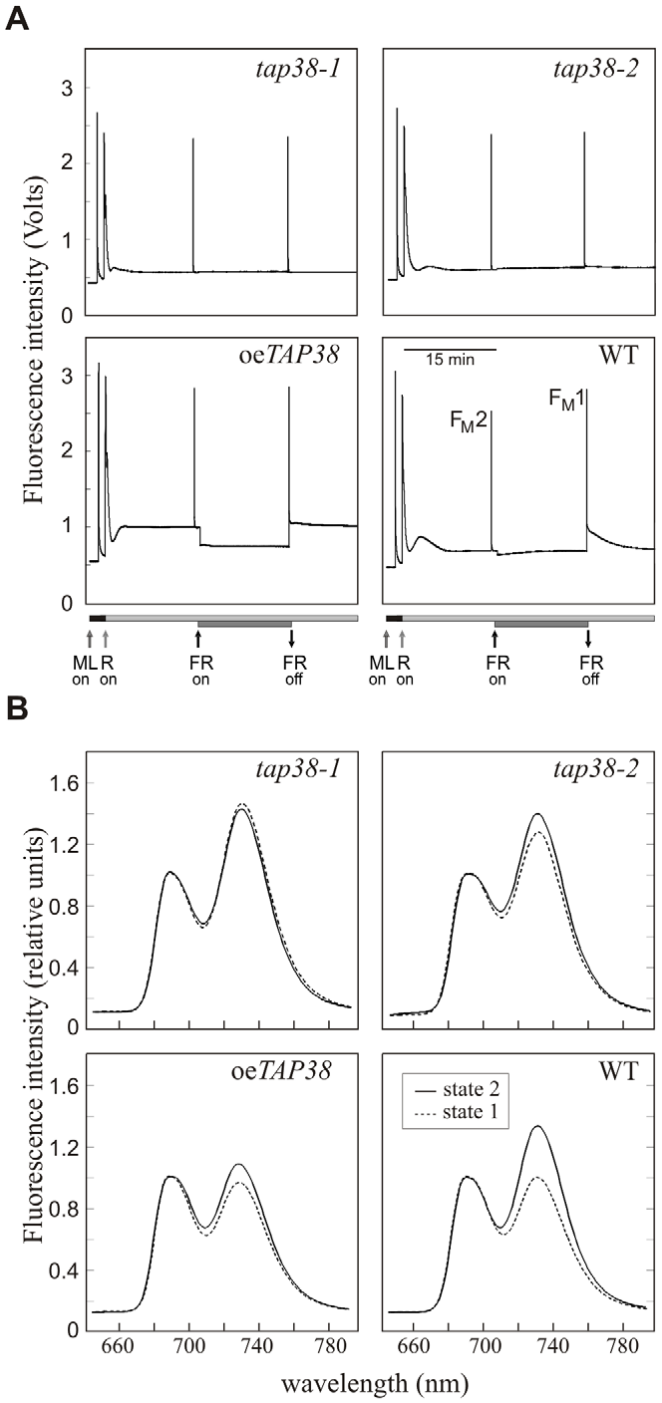
TAP38 is required for state transitions. (A) Red light (R) and red light supplemented with far-red (FR) light were used to induce transitions to state 2 and state 1, respectively. F_M_1 and F_M_2 represent maximal chlorophyll fluorescence levels in states 1 and 2, respectively. Horizontal bars indicate the length of illumination. Arrows point to the moment when the specific light is switched on/off. Traces are the average of 10 replicates. ML, measuring light. (B) Low-temperature (77 K) fluorescence emission spectra of thylakoids were recorded after exposure of plants to light inducing either state 1 (dashed lines, far-red light of 740 nm) or state 2 (solid lines, low light; 80 µmol m^−2^ s^−1^) (see also Materials and Methods). The excitation wavelength was 475 nm, and spectra were normalized with reference to peak height at 685 nm. Traces are the average of 10 replicates.

To determine whether TAP38 is involved in state transitions, chlorophyll fluorescence was measured in WT, *tap38*, and oe*TAP38* leaves (Figure 4A). Plants were exposed to light conditions that stimulate either state 2 (red light) or state 1 (far-red light) [35],[36], and the corresponding maximum fluorescence in state 2 (F_M_2) and in state 1 (F_M_1) values were determined. Because the light intensity chosen to induce state transitions did not elicit photoinhibition (as monitored by measurements of the maximum quantum yield [F_V_/F_M_]), changes in F_M_, the maximum fluorescence, could be attributed to state transitions alone. This allowed us to calculate the degree of quenching of chlorophyll fluorescence due to state transitions (qT) [36]. In the *tap38* mutants, qT was markedly decreased (*tap38-1*, 0.01±0.003; *tap38-2*, 0.03±0.001; WT, 0.10±0.001). In *tap38-1* plants complemented with the *TAP38* genomic sequence (including its native promoter), qT values were normal, confirming that state transitions require TAP38. Interestingly, oe*TAP38* plants exhibited qT values of about 0.01±0.001, indicating that both absence and excess of TAP38 interfere with the ability to undergo reversible state transitions.

To determine the antenna sizes of PSII and PSI, 77K fluorescence emission spectra were measured under state 1 (exposure to far-red light) and state 2 (low light) conditions as described [11],[12],[37] (Figure 4B). The spectra were normalized at 685 nm, the peak of PSII fluorescence. In WT, the transition from state 1 to state 2 was accompanied by a marked increase in relative PSI fluorescence at 730 nm, reflecting the redistribution of excitation energy from PSII to PSI. In contrast, in *tap38* leaves, the PSI fluorescence peak was relatively high even under state 1-promoting conditions, implying that the mutants were blocked in state 2—i.e., pLHCII should be predominantly attached to PSI. Additionally, under state 2-promoting light conditions, the PSI antenna size (expressed as F_730_/F_685_) was larger in *tap38* mutants than in WT (*tap38-1*, 1.47; *tap38-2*, 1.45; WT, 1.38; see also Table S1), arguing in favor of the idea that in *tap38* plants, a larger fraction of the mobile pool of LHCII can attach to PSI. On the contrary, in oe*TAP38* plants, the relative fluorescence of PSI hardly increased at all under conditions expected to induce the state 1→state 2 shift (Figure 4B; Table S1). This behavior resembles that of *stn7* mutants, which are blocked in state 1, i.e., with LHCII permanently attached to PSII [12].

### Levels of LHCII Phosphorylation Correlate Inversely with TAP38 Concentrations

**Figure 5:**
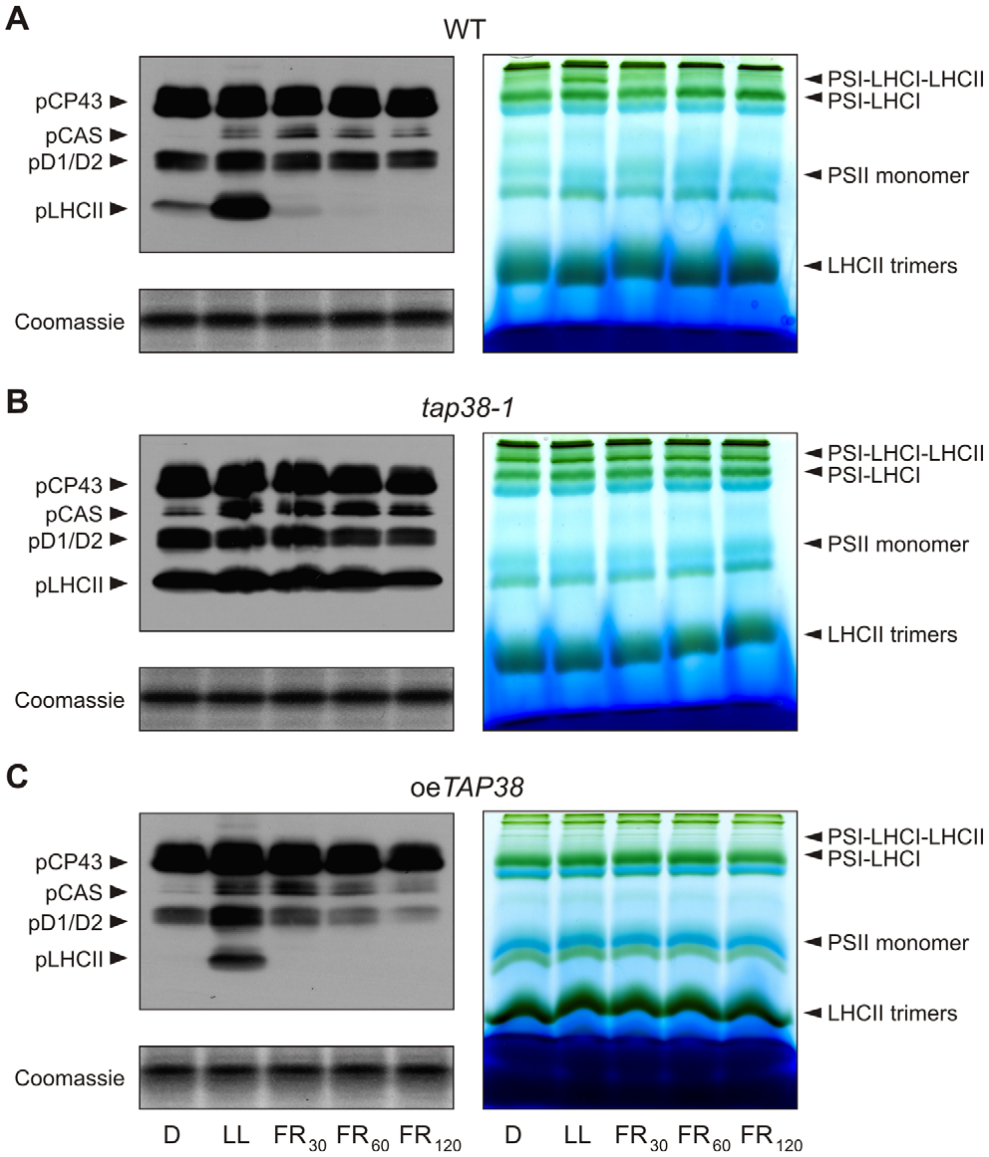
Levels of LHCII phosphorylation correlate inversely with TAP38 concentrations. Left panel, thylakoid proteins extracted from WT (A), *tap38-1* (B), and oe*TAP38* (C) plants kept in the dark (D; state 1), subsequently exposed to low light (LL; state 2), and then to far-red light for 30, 60, and 120 min (FR_30_, FR_60_, FR_120_; state 1) were fractionated by SDS-PAGE. Phosphorylation of LHCII and PSII core proteins was detected by immunoblot analysis with a phosphothreonine-specific antibody. One out of three immunoblots for each genotype is shown. pCAS, phosphorylated CAS [44]; pCP43, phosphorylated CP43; pD1/D2, phosphorylated PSII-D1/D2; pLHCII, phosphorylated LHCII; Coomassie, portion of Coomassie-stained PA gels, identical to the ones blotted and corresponding to the LHCII migration region, were used as loading control. Right panel, thylakoid proteins of WT (A), *tap38-1* (B), and oe*TAP38* (C) plants treated as in the left panel were subjected to BN-PAGE analysis. Accumulation of the state 2-associated 670-kDa protein complex [14] correlates with the phosphorylation level of LHCII. Note that *tap38-2* behaved very similarly to *tap38-1* (data not shown). One out of three BN-PAGEs for each genotype is shown.

It is generally accepted that state transitions require reversible phosphorylation of LHCII [2],[4],[5]. Therefore, the phosphorylation state of LHCII was monitored under light conditions that favor state 1 (dark or far-red light treatment) or state 2 (low light). Plants with abnormal levels of TAP38, and WT plants were dark adapted for 16 h (state 1), then exposed to low light (80 µmol m^−2^ s^−1^, 8 h) (state 2), and then to far-red light (4.5 µmol m^−2^ s^−1^, 740 nm) for up to 120 min to induce a return to state 1. Thylakoid proteins were isolated after each treatment, fractionated by sodium dodecyl sulfate (SDS)-PAGE, and analyzed with a phosphothreonine-specific antibody (Figure 5, left panels). WT plants showed the expected increase in pLHCII during the transition from state 1 (dark [D]) to state 2 (low light [LL]), followed by a progressive decrease in pLHCII upon exposure to far-red light (FR). In *tap38* mutants, levels of pLHCII were aberrantly high at all time points, whereas the oe*TAP38* plants again mimicked the *stn7* phenotype [9],[12], displaying constitutively reduced levels of pLHCII.

### Quantification of PSI-LHCI-LHCII Complex Formation under Varying TAP38 Concentrations

**Figure 6:**
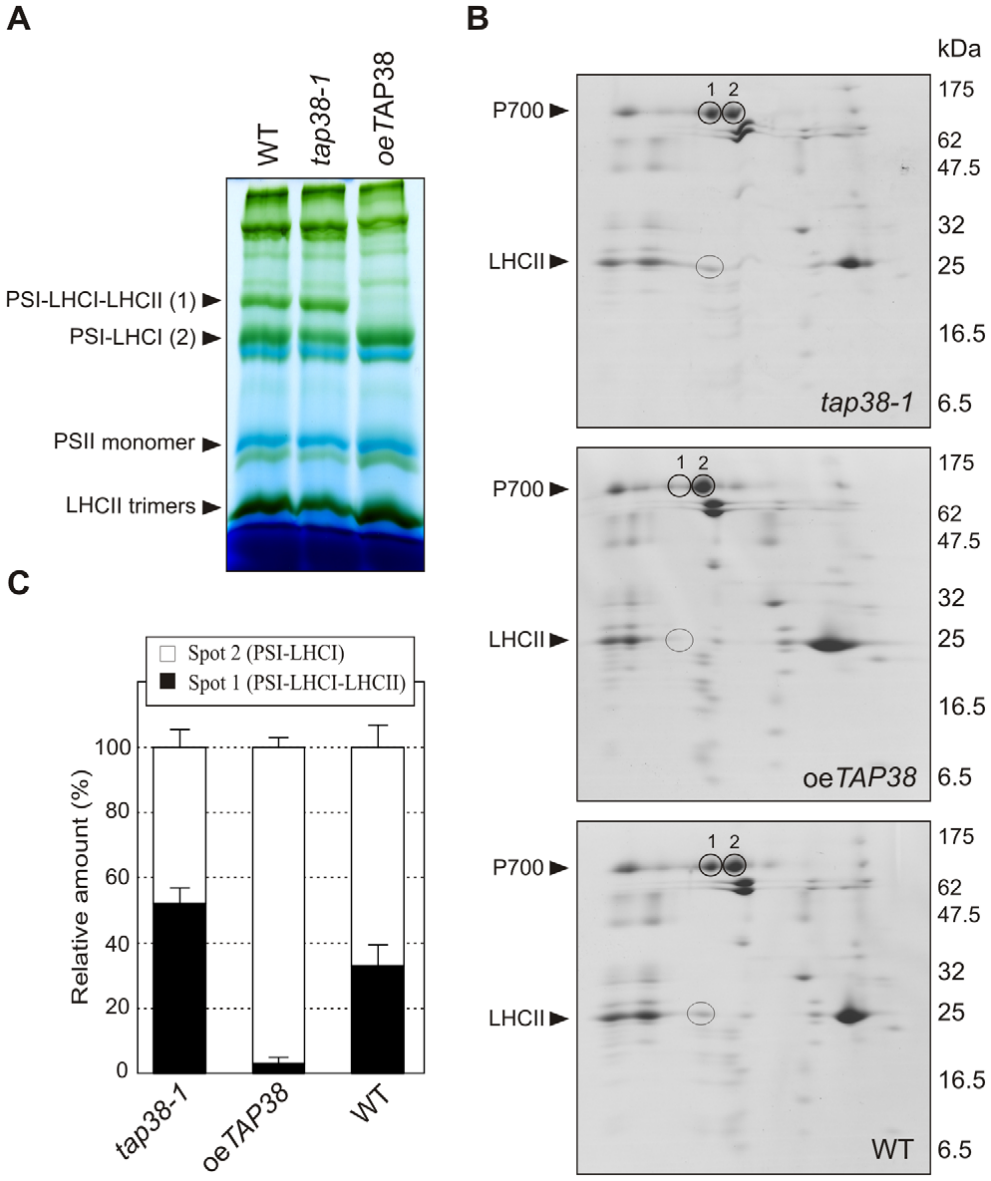
Quantification of PSI-LHCI and PSI-LHCI-LHCII complexes under state 2 conditions. (A) BN-PAGE of identical amounts of thylakoid proteins from WT, *tap38-1*, and oe*TAP38* plants adapted to state 2 (low light; 80 µmol m^−2^ s^−1^). Bands representing the PSI-LHCI-LHCII (1) and PSI-LHCI (2) complexes are indicated. The differences in the separation behavior of the BN-gel in comparison to the ones in Figure 5 are caused by the longer electrophoresis running time. (B) The WT, *tap38-1*, and oe*TAP38* lanes from the BN-PAGE in (A) were fractionated further by denaturing 2D-PAGE. Gels were stained with Coomassie Blue. LHCII, light-harvesting complex of PSII (the bands indicative for the PSI-LHCI-LHCII (1) and PSI-LHCI (2) complexes are encircled); P700, photosystem I reaction center. (C) Densitometric quantification of the spots representing PSI-LHCI-LHCII (spot 1) and PSI-LHCI (spot 2) in (B). Values are averages of three independent 2D gels for each genotype. Bars indicate standard deviations. Note that *tap38-2* behaves very similarly to *tap38-1* (data not shown).

To directly visualize how alterations in LHCII phosphorylation in lines lacking or overexpressing TAP38 affect the distribution of the mobile LHCII fraction between the two photosystems, we subjected thylakoid protein complexes of plants adapted to state 1 (dark and far-red light treatments) or state 2 (low-light treatment) to nondenaturing Blue-native (BN) PAGE [38] (Figure 5, right panels). In this assay, a pigment–protein complex of about 670 kDa, which represents pLHCII associated with the PSI-LHCI complex [14],[38],[39], can be visualized. Whereas in WT thylakoids, the 670-kDa complex was only observable under state 2 conditions (Figure 5A, right panel), as previously reported [14],[39]; the constitutive phosphorylation of LHCII in the *tap38* mutants was associated with the presence of a prominent band for the 670-kDa complex under all light conditions (Figure 5B, right panel). The formation of the 670-kDa complex was totally prevented in oe*TAP38* plants with a block in state 1 and highly reduced levels of pLHCII (Figure 5C, right panel). Two-dimensional (2D) PA gel fractionation confirmed that the pigment–protein complex consists of PSI and LHCI subunits, together with a portion of pLHCII that associates with PSI upon state 1→state 2 transition in WT plants (Figure 6; [14]). Additionally, quantification of the different PSI complexes on 2D PA gels showed that the number of PSI complexes associated with LHCII was increased in the *tap38* mutants (Figures 6B and 6C), supporting the findings obtained from the 77K fluorescence analyses.

### Recombinant TAP38 Is Able to Directly Dephosphorylate pLHCII

**Figure 7:**
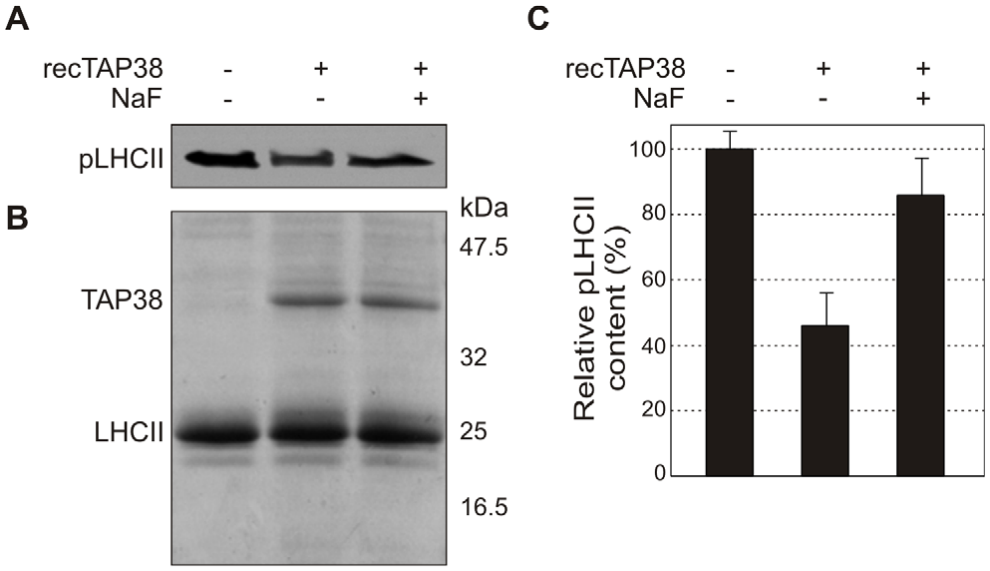
Recombinant TAP38 directly dephosphorylates pLHCII in vitro. (A) Equal amounts of pLHCII isolated from *tap38-1* mutant plants, treated with or without recombinant TAP38, were separated by SDS-PAGE and immunodecorated with phosphothreonine-specific antibodies. NaF (10 mM) was added to specifically inhibit phosphatase activity. (B) A replicate gel of the samples as in (A) was stained with Coomassie Blue as a loading control. The recombinant TAP38 protein and LHCII bands are shown. (C) Densitometric quantification of the bands in (A), representing the phosphorylation levels of LHCII under the different conditions.

An in vitro dephosphorylation assay was established to assess the capability of TAP38 to directly dephosphorylate pLHCII. To this purpose, an N-terminal His-tag fusion of the TAP38 phosphatase was expressed in *Escherichia coli* and purified (see Materials and Methods). Solubilized thylakoids from *tap38-1* mutant plants were then fractionated by sucrose gradient ultracentrifugation, and the protein fraction enriched in pLHCII was isolated. Subsequently, the pLHCII pigment–protein complex was incubated at 30°C for 2 h either in the presence or absence of the recombinant TAP38 phosphatase. At the end of the incubation period, the reaction mixture was fractionated by SDS-PAGE and subjected to immunoblotting using a phosphothreonine-specific antibody (Figure 7). Clearly, the addition of the recombinant TAP38 decreased the level of LHCII phosphorylation by about 50% (relative to the untreated pLHCII sample). In the presence of the phosphatase inhibitor NaF, TAP38 addition did not markedly alter the phosphorylation level of LHCII. Taken together, these findings suggest that TAP38 is able to directly dephosphorylate pLHCII.

### Plants without TAP38 Show Improved Photosynthesis and Growth under Low Light

**Figure 8:**
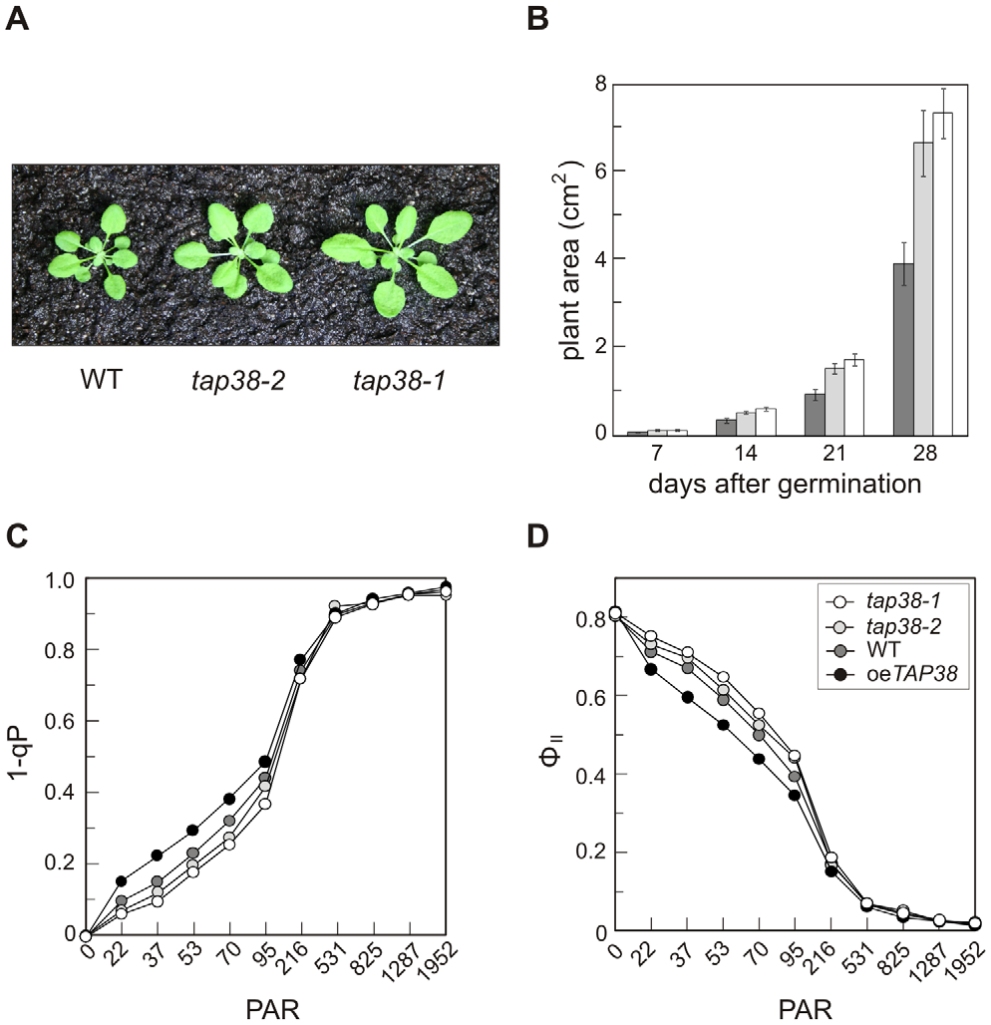
Growth characteristics and photosynthetic performance of *tap38* mutant plants. (A) Phenotypes of 4-wk-old *tap38-1*, *tap38-2*, and WT plants grown under low-light conditions (80 µmol m^−2^s^−1^) on a 12 h/12 h light/dark regime. (B) Growth curve. Leaf areas of 20 plants of each genotype (WT, grey bars; *tap83-1*, white bars; *tap38-2*, light grey bars) were measured over a period of 4 wk after germination. Mean values ± standard deviations (SDs; bars) are shown. (C and D), Measurements of light dependence of the photosynthetic parameter 1-qP (C) and effective quantum yield of PSII (ΦII; [D]) of plants grown as in (A). WT, filled grey circles; *tap38-1*, open circles; *tap38-2*, filled light-grey circles; oe*TAP38*, filled black circles; PAR, photosynthetically active radiation in µmol m^−2^ s^−1^. Average values were determined from five independent measurements (SD<5%).

When kept under low-light intensities (80 µmol m^−2^ s^−1^) that favor state 2, *tap38* mutants grew larger than WT plants (Figure 8A), whereas oe*TAP38* plants behaved like WT (unpublished data). Detailed growth measurements revealed that the *tap38* mutants exhibited a constant growth advantage over WT plants, starting at the cotyledon stage (Figure 8B). Because this difference might be attributable to altered photosynthetic performance, parameters of thylakoid electron flow were measured. The fraction of Q_A_ (the primary electron acceptor of PSII) present in the reduced state (1-qP) was lower in *tap38-1* (0.06±0.01) and *tap38-2* plants (0.07±0.01) than in WT (0.10±0.01), when both genotypes were grown as in Figure 8A and chlorophyll fluorescence was excited with 22 µmol m^−2^ s^−1^ actinic red light. Comparable differences in the redox state of the primary electron acceptor persisted up to 95 µmol m^−2^ s^−1^ actinic red light (Figure 8C), indicating that the *tap38* mutants can redistribute a larger fraction of energy to PSI, in accordance with the increase in its antenna size under state 2 light conditions (see Figure 4B; Table S1 and Figure 6). This idea was supported by measurements of the maximum (F_V_/F_M_) and effective (Φ_II_) quantum yields of PSII. F_V_/F_M_ remained unaltered in mutant plants (see Figure 8D, dark-adapted plants, photosynthetically active radiation [PAR] = 0), indicating WT-like efficiency of mutant PSII complexes. However, Φ_II_ was increased in *tap38-1* (0.75±0.01) and *tap38-2* (0.73±0.02) relative to WT (0.72±0.01), suggesting that electron flow through the thylakoids was more efficient in *tap38* mutants (Figure 8D). The improvement in photosynthetic performance of the *tap38* mutants was most pronounced under low and moderate illumination (Figures 8C and 8D), as expected from their growth phenotype.

## Discussion

### Possible Modes of Action of TAP38

How does TAP38 control LHCII dephosphorylation? Three possibilities appear plausible: TAP38 (1) negatively regulates the activity of STN7 (e.g., by dephosphorylating it [40]), (2) dephosphorylates LHCII directly, or (3) forms part of a phosphorylation/dephosphorylation cascade that controls the activity of the LHCII kinase or phosphatase. The observation that oe*TAP38* plants, although showing a >20-fold increase in TAP38 levels, still exhibit residual LHCII phosphorylation (see Figure 5C), argues against the idea that TAP38 does inhibit STN7 by dephosphorylation. Differences in TAP38 levels resulted in a clear change in pLHCII levels: although in *tap38* mutants a strong reduction in TAP38 led to a constantly high level of pLHCII and an increase in the amount of the PSI-LHCI-LHCII complex, strong overexpression of TAP38 (oe*TAP38*) caused the complete disappearance of pLHCII attached to PSI, although pLHCII was still present.

Taking these observations together, it appears that the TAP38 phosphatase acts specifically on pLHCII associated to PSI-LHCI complexes. Indeed, the dephosphorylation of pLHCII still attached to PSII under state 2-inducing light conditions seems unfavorable in terms of energy efficiency.

Interestingly, in WT where pLHCII levels can vary dramatically depending on the light conditions [9],[12] (see also Figure 5A), TAP38 seems to be constitutively expressed under the different light conditions applied (see Figure 3C). A plausible explanation for this is that TAP38 is constitutively active and directly responsible for the dephosphorylation of pLHCII. For that, TAP38 would need to be present in a certain concentration range (as it is the case for WT) to constantly dephosphorylate pLHCII. In agreement with that, thylakoid protein phosphatase reactions have been described as redox independent, leading to the conclusion that the redox dependency of LHCII phosphorylation is a property of the kinase reaction [41]. This, together with the observation that Stt7 levels increase under prolonged state 2 conditions (favoring LHCII phosphorylation) and decrease under state 1 conditions (favoring dephosphorylation of LHCII) [17], argues in favor of the hypothesis that the LHCII kinase is the decisive factor in controlling the phosphorylation state of LHCII. Despite the obvious TAP38 dosage dependence of pLHCII dephosphorylation (see Figures 5 and 6), TAP38 activity could be regulated on other levels than only its abundance. However, the strong decrease or increase of TAP38 levels in *tap38* mutant and oe*TAP38* plants might interfere with other types of regulation in these genotypes.

### Is TAP38 the Long-Sought LHCII Phosphatase?

As outlined above, the dependence of LHCII dephosphorylation upon TAP38 dosage—when comparing *tap38* mutants, WT, and TAP38 overexpressors—strongly suggests that TAP38 dephosphorylates pLHCII directly, particularly when it is associated with the PSI-LHCI complex. Alternatively, TAP38 could act in a phosphorylation/dephosphorylation cascade that controls the activity of the LHCII phosphatase. Although the latter hypothesis cannot be totally excluded, a set of evidences point to a direct role of TAP38 on LHCII phosphorylation. Indeed, our in vitro dephosphorylation assay clearly indicated that TAP38 can dephosphorylate pLHCII directly (see Figure 7). Moreover, as in the case of STN kinases, extensive efforts searching to identify other LHCII phosphatase candidates failed: knockout lines for all the protein phosphatases demonstrated to be located in the chloroplast [25]–[29] did not show any alteration in LHCII phosphorylation. Additionally, extensive biochemical studies did not reveal the existence of a complex network of phosphatases involved in LHCII dephosphorylation, but postulated the involvement of only two distinct chloroplast protein phosphatases from different families in the dephosphorylation of thylakoid phosphoproteins [20]–[23],[42]. Our data support this notion, as shown by the absence of major alterations in the phosphorylation pattern of CP43, D1, and D2 subunits in *tap38* mutant plants (see Figure 5). Moreover, pLHCII dephosphorylation was suggested to be catalyzed by only two independent protein phosphatases, a membrane-bound one and a stromal protein phosphatase [42]. In contrast to this, our results clearly show that TAP38, a thylakoid-associated phosphatase, alone is responsible for LHCII dephosphorylation. Thus, although slightly leaky, the *tap38-1* mutants show a large fraction of LHCII in the phosphorylated state under all investigated conditions (see Figure 5). If a second LHCII phosphatase with redundant function would operate in chloroplasts, one would expect some residual dephosphorylation of pLHCII. A plausible explanation for the previously shown stromal pLHCII dephosphorylation activity [22] might be that during the preparation of stromal extracts, a significant portion of TAP38 was released from the thylakoid membrane into the stroma. Interestingly, TAP38 appears to influence also the phosphorylation levels of other thylakoid proteins, as shown by the higher phosphorylation of the CAS protein in *tap38-1* thylakoids (see Figure 5). Taking these observations together, it appears that, as in the case of the STN kinases, two distinct phosphatases are needed to dephosphorylate LHCII and PSII core proteins. TAP38, similar to the STN7 kinase, seems to have a high specificity for pLHCII associated with PSI-LHCI complexes as substrate. The counterpart of STN8 [9],[43], the PSII core–specific phosphatase, remains to be identified. However, as in the case of the STN7 and STN8 kinases, some degree of substrate overlap seems to exist also between the phosphatases, as shown by the more rapid dephosphorylation of PSII-D1/D2 subunits in the *TAP38* overexpressor lines exposed to far-red light conditions (see Figure 5C). Additionally, it is noteworthy that the activity of TAP38 does not seem to be restricted to STN7 substrates, as shown by its influence on CAS protein phosphorylation, previously reported to be a substrate of the STN8 kinase [44].

### Uncoupling of LHCII Phosphorylation from PQ Redox State

It is known that an increase in the relative size of the reduced fraction of the plastoquinone pool (PQH_2_) enhances phosphorylation of LHCII [1],[5],[39],[45]. Depletion of TAP38 in *tap38* mutants, however, increases both LHCII phosphorylation (see Figure 5B) and PQ oxidation (see 1-qP values in Figure 8C). This discrepancy can be resolved by assuming that the enhanced oxidation of PQ caused by the increase in PSI antenna size (and LHCII phosphorylation) in *tap38* plants is not sufficient to down-regulate the LHCII kinase to such an extent that it can compensate for the decline in LHCII dephosphorylation.

### How Can Absence of TAP38 Improve Photosynthesis and Growth?

The enhanced photosynthetic performance indicated by an increase in Φ_II_ and a decrease of 1-qP (see Figure 8C and 8D), as well as the growth advantage of the *tap38* mutants under constant moderate-light intensities that stimulate LHCII phosphorylation and state 2, can be attributed to the redistribution of a larger fraction of energy to PSI. This is in accordance with the increase in PSI antenna size in *tap38* mutants when compared to WT plants (see Figure 4B, Table S1, and Figure 6). Therefore, it is straightforward to speculate that the enhanced PSI antenna size provides the *tap38* mutants with a more robust photosynthetic electron flow under conditions that preferentially excite PSII and induce state 2. As a consequence of the more balanced light reaction, the photosynthetic efficiency is improved resulting in an increased growth rate. However, the fitness advantage will revert under conditions that induce state 1, or under more natural conditions with fluctuating light; here, it can be expected that *tap38* mutants will perform less efficiently than the WT with respect to photosynthesis and growth, very similar to what has been observed for the *stn7* mutant [12],[13].

### Outlook

Taken together, future analyses should clarify which protein phosphatase is involved in the dephosphorylation of PSII core proteins and which are the counterparts of higher plant phosphatases, including TAP38, in *Chlamydomonas* (which apparently lacks a *TAP38* ortholog). Additionally, further biochemical evidences that TAP38 (and STN7) uses pLHCII as a substrate will be very important for the complete molecular dissection of state transitions.

## Materials and Methods

### Plant Material and Growth Measurements

Procedures for plant propagation and growth measurements have been described elsewhere [46]. The *tap38-2* insertion line (SALK_025713) was identified in the SALK collection [34] (http://signal.salk.edu/), whereas insertion line *tap38-1* (SAIL_514_C03) originated from the Sail collection [33]. Both lines were identified by searching the insertion flanking database SIGNAL (http://signal.salk.edu/cgi-bin/tdnaexpress). To generate oe*TAP38* lines, the coding sequence of *TAP38* was cloned into the plant expression vector pH2GW7 (Invitrogen). For complementation of the *tap38-1* mutant, the *TAP38* genomic DNA, together with 1 kb of its natural promoter, was ligated into the plant expression vector pP001-VS. The constructs were used to transform flowers of Col-0 or *tap38-1* mutant plants by the floral dipping technique as described [47]. Transgenic plants, after selection for resistance to hygromycin (oe*TAP38*) or Basta herbicide (complemented *tap38-1*), were grown on soil in a climate chamber under controlled conditions (PAR: 80 µmol m^−2^ s^−1^, 12/12 h dark/light cycles). The T2 generation of the oe*TAP38* plants was used for the experiments reported. Successful complementation of *tap38-1* mutants was confirmed by measurements of chlorophyll fluorescence and LHCII phosphorylation levels under light regimes promoting state transitions.

### Subcellular Localization of the TAP38-dsRED Fusion in *Arabidopsis* Protoplasts

The full-length coding region of the *TAP38* gene was cloned into the vector pGJ1425, in frame with, and immediately upstream of the sequence encoding dsRED [32]. Isolation, transfection, and fluorescence microscopy of *A. thaliana* protoplasts were performed as described [48].

### In Vitro Import of TAP38 into Pea Chloroplasts

The coding region of *TAP38* was cloned into the pGEM-Teasy vector (Promega) downstream of its SP6 promoter region, and mRNA was produced in vitro using SP6 RNA polymerase (MBI Fermentas). The TAP38 precursor protein was synthesized in a Reticulocyte Extract System (Flexi; Promega) in the presence of [^35^S]methionine. Aliquots of the translation reaction were incubated with intact chloroplasts, and protein uptake was analyzed after treatment of isolated chloroplasts with thermolysin (Calbiochem) as described previously [49]. Labeled proteins were subjected to SDS-PAGE and detected by phosphorimaging (Typhoon; Amersham Biosciences).

### cDNA Synthesis, Semiquantitative Reverse-Transcriptase PCR, and Real-Time PCR

Total RNA was extracted with the RNeasy Plant Mini Kit (QIAGEN) according to the manufacturer's instructions. cDNA was prepared from 1 µg of total RNA using the iScript cDNA Synthesis Kit (Bio-Rad) according to the manufacturer's instructions. For semiquantitative reverse-transcriptase PCR, cDNA was diluted 10-fold, and 3 µl of the dilution was used in a 20-µl reaction. Thermal cycling consisted of an initial step at 95°C for 3 min, followed by 30 cycles of 10 s at 95°C, 30 s at 55°C, and 10 s at 72°C. For real-time PCR analysis, 3 µl of the diluted cDNA was mixed with iQ SYBR Green Supermix (Bio-Rad). Thermal cycling consisted of an initial step at 95°C for 3 min, followed by 40 cycles of 10 s at 95°C, 30 s at 55°C, and 10 s at 72°C, after which a melting curve was performed. Real-time PCR was monitored using the iQ5Multi-Color Real-Time PCR Detection System (Bio-Rad). All reactions were performed in triplicate with at least two biological replicates.

### Protein Isolation and Immunoblot Analysis

Total protein extracts and proteins from total chloroplasts, thylakoids, and the stroma fraction were prepared from 4-wk-old leaves in the presence of 10 mM NaF as described [48],[50]. Immunoblot analyses with phosphothreonine-specific antibodies (Cell Signaling) or polyclonal antibodies raised against the mature TAP38 protein were performed as described [45].

### Blue-Native (BN)-PAGE and 2D Polyacrylamide Gel Electrophoresis (2D-PAGE)

For BN-PAGE, thylakoid membranes were prepared as described above. Aliquots corresponding to 100 µg of chlorophyll were solubilized in solubilization buffer (750 mM 6-aminocaproic acid; 5 mM EDTA [pH 7]; 50 mM NaCl; 1.5% digitonin) for 1 h at 4°C. After centrifugation for 1 h at 21,000*g*, the solubilized material was fractionated by nondenaturing BN-PAGE at 4°C as described [38].

For 2D-PAGE, samples were fractionated in the first dimension by BN-PAGE as described above and subsequently by denaturing SDS-PAGE as described previously [51]. Densitometric analysis of the stained gels was performed using the Lumi Analyst 3.0 (Boehringer).

### Measurements of State Transitions and 77 K Fluorescence

State transitions were measured by pulse amplitude modulation fluorometry (PAM) [35],[36] and 77 K fluorescence emission analysis [12],[37]. Plants adapted to state 1 conditions were obtained by incubation either in darkness or far-red light, whereas state 2 was induced by either red- or low-light illumination. Both state 1 and state 2 light-inducing conditions were used in different combinations, since they resulted in identical effects on state transitions. Additionally, there was no major reason to prefer one light setting to the other, except for the fact that the PAM fluorometer is equipped with red and far-red lights. For state transition measurements, five plants of each genotype were analyzed, and mean values and standard deviations were calculated. In vivo chlorophyll *a* fluorescence of single leaves was measured using the Dual-PAM 100 (Walz). Pulses (0.5 s) of red light (5,000 µmol m^−2^ s^−1^) were used to determine the maximum fluorescence and the ratio (F_M_−F_0_)/F_M_ = F_V_/F_M_. Quenching of chlorophyll fluorescence due to state transitions (qT) was determined by illuminating dark-adapted leaves with red light (35 µmol m^−2^ s^−1^, 15 min) and then measuring the maximum fluorescence in state 2 (F_M_2). Next, state 1 was induced by adding far-red light (maximal light intensity corresponding to level 20 in the Dual-PAM setting, 15 min), and recording F_M_1. qT was calculated as (F_M_1−F_M_2)/F_M_1 [36].

For 77 K fluorescence emission spectroscopy, the fluorescence spectra of thylakoids were recorded after irradiating plants with light that favored excitation of PSII (80 µmol m^−2^ s^−1^, 8 h) or PSI (LED light of 740 nm wavelength, 4.6 µmol m^−2^ s^−1^, 2 h). Thylakoids were isolated in the presence of 10 mM NaF as described [11], and 77 K fluorescence spectra were obtained by excitation at 475 nm using a Spex Fluorolog mod.1 fluorometer (Spex Industries). The emission between 600 and 800 nm was recorded, and spectra were normalized relative to peak height at 685 nm. Data frequency was of 0.5 nm with an integration time of 0.1 s.

### In Vitro Dephosphorylation Assay

pLHCII was obtained from fractionation of *tap38-1* thylakoids by sucrose gradient ultracentrifugation as previously described [45]. The cDNA sequence of mature TAP38 was cloned into pET151 (Invitrogen), and recombinant TAP38 (recTAP38) was expressed in the *E. coli* strain BL21 with a N-terminal-6x His-tag. recTAP38 was purified under denaturing conditions following a Ni-NTA batch purification procedure according to the manufacturer's instructions (Qiagen). After protein precipitation in 10% trichloroacetic acid (TCA) followed by three washing steps with absolute ethanol, around 500 µg of TAP38 protein were resuspended in 500 µl of 1% (w/v) lithium dodecyl sulfate (LDS), 12.5% (w/v) sucrose, 5 mM ε-aminocaproic acid, 1 mM benzamidine, and 50 mM HEPES KOH (pH 7.8), as previously described [52]. Subsequently, TAP38 protein was boiled for 2 min at 100°C and then transferred for 15 min at 25°C. Then, dithiothreitol (DTT; 75 mM final concentration) was added, and the solution was subjected to three freezing-thawing cycles (20 min at −20°C, 20 min at −80°C, 20 min at −20°C, thawing in a ice-water bath, and 5 min at 25°C). After completion of the three freezing-thawing cycles, octyl-glucopyranoside (OGP; 1% [w/v] final concentration) was added, and the solution was kept on ice for 15 min. Afterwards, KCl (75 mM, final concentration) was added to precipitate the LDS detergent. After centrifugation at 16,000*g* at 4°C for 10 min, the supernatant containing the refolded TAP38 in the presence of 1% (w/v) OGP was collected. Subsequently, 1 µl of phosphatase was incubated together with pLHCII corresponding to 2 µg of total chlorophyll. The dephosphorylation reaction was performed in 50 µl containing 0.06% (w/v) dodecyl-ß-D-maltoside, 5 mM Mg-acetate, 5 mM DTT, 100 mM HEPES (pH 7.8), at 37°C for 2 h as previously described [22]. The reaction mixture was loaded on a SDS-PAGE and immunodecorated with a phosphothreonine-specific antibody, as described above.

## Supporting Information

Figure S1 **Insertion alleles of** ***At4g27800*** **and their effects on splice variant expression.** (A) T-DNA insertions in the *At4g27800* locus. The different coding sequences of the three splice variants are depicted as grey boxes. The respective 5′ and 3′ UTRs are shown in white. Introns are indicated as thin lines. Splice variants *At4g27800.1* (*TAP38*) and *At4g27800.3* can be distinguished due to an insertion of four additional nucleotides in exon 9 of *At4g27800.3* leading to a stop codon. Arrows (not drawn to scale) indicate the positions of primer pairs used in PCR analysis. Sequences of primers indicated as 1, 2, 3, and 4 are: *At4g27800.1/TAP38-At4g27800.2*-specific primer (No. 1): 5′-ACATGGGAATGTGCAGCTTG; *At4g27800.1/TAP38-At4g27800.2-At4g27800.3* (No. 2): 5′-GTGAAGACATCCATATGCCA; *At4g27800.2*-specific primer (No. 3): 5′-AATACCCTCCTCAGCCTTTC; *At4g27800.3*-specific primer (No. 4): 5′-ACATGGGAATGTGCAGGCAA. (B) Semiquantitative reverse transcriptase (RT)-PCR analysis to verify the presence of the three splice variants in *Arabidopsis* WT leaves. Primer combinations employed in RT-PCR reactions are numbered as in (A). *Ubiquitin* (*UBI*) was amplified as a control for equal loading (Ubiquitin forward primer: 5′-GGAAAAAGGTCTGACCGACA; Ubiquitin reverse: 5′-CTGTTCACGGAACCCAATTC). Aliquots (10 µl) of representative semiquantitative RT-PCR reactions (30 cycles) were electrophoresed on a 2% (w/v) agarose gel to differentiate between *At4g27800.1* (*TAP38*) and *At4g27800.2*. Note that for the *At4g27800.3* splice variant, no signal could be obtained.
(0.35 MB TIF)

Table S1 **Energy distribution between PSI and PSII measured as the fluorescence emission ratio at 730 nm and 685 nm (F_730_/F_685_).**
(0.04 MB DOC)

## Abbreviations

F_M_1: maximum fluorescence in state 1
F_M_2: maximum fluorescence in state 2
F_V_/F_M_: the maximum quantum yield of photosystem II
Φ_II_: effective quantum yield of photosystem II
PAR: photosynthetically active radiation
PSI: photosystem I
PSII: photosystem II
qT: the degree of quenching of chlorophyll fluorescence due to state transitions
WT: wild type
