# Supplementary figures and images for: Role of Plastid Protein Phosphatase TAP38 in LHCII Dephosphorylation and Thylakoid Electron Flow
Source: PLoS Biol. 2010 Jan 26;8(1):e1000288. doi: 10.1371/journal.pbio.1000288 (PMC2811158; doi:10.1371/journal.pbio.1000288)

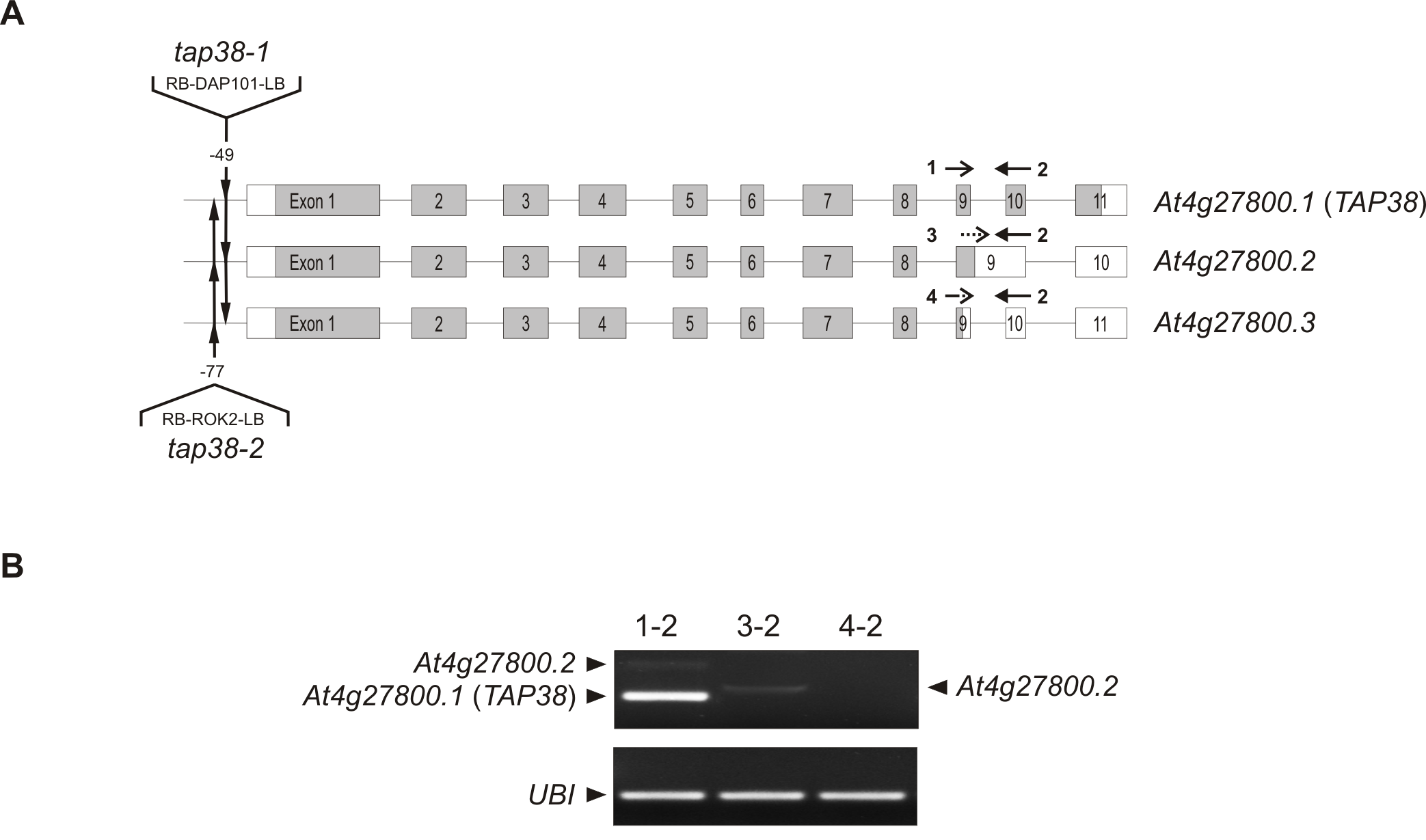

Supplement: Figure S1 — Insertion alleles of At4g27800 and their effects on splice variant expression. (A) T-DNA insertions in the At4g27800 locus. The different coding sequences of the three splice variants are depicted as grey boxes. The respective 5′ and 3′ UTRs are shown in white. Introns are indicated as thin lines. Splice variants At4g27800.1 (TAP38) and At4g27800.3 can be distinguished due to an insertion of four additional nucleotides in exon 9 of At4g27800.3 leading to a stop codon. Arrows (not drawn to scale) indicate the positions of primer pairs used in PCR analysis. Sequences of primers indicated as 1, 2, 3, and 4 are: At4g27800.1/TAP38-At4g27800.2-specific primer (No. 1): 5′-ACATGGGAATGTGCAGCTTG; At4g27800.1/TAP38-At4g27800.2-At4g27800.3 (No. 2): 5′-GTGAAGACATCCATATGCCA; At4g27800.2-specific primer (No. 3): 5′-AATACCCTCCTCAGCCTTTC; At4g27800.3-specific primer (No. 4): 5′-ACATGGGAATGTGCAGGCAA. (B) Semiquantitative reverse transcriptase (RT)-PCR analysis to verify the presence of the three splice variants in Arabidopsis WT leaves. Primer combinations employed in RT-PCR reactions are numbered as in (A). Ubiquitin (UBI) was amplified as a control for equal loading (Ubiquitin forward primer: 5′-GGAAAAAGGTCTGACCGACA; Ubiquitin reverse: 5′-CTGTTCACGGAACCCAATTC). Aliquots (10 µl) of representative semiquantitative RT-PCR reactions (30 cycles) were electrophoresed on a 2% (w/v) agarose gel to differentiate between At4g27800.1 (TAP38) and At4g27800.2. Note that for the At4g27800.3 splice variant, no signal could be obtained. (0.35 MB TIF) [file pbio.1000288.s001.tif]
