# Supplementary material for: Role of Plastid Protein Phosphatase TAP38 in LHCII Dephosphorylation and Thylakoid Electron Flow
Source: PLoS Biol. 2010 Jan 26;8(1):e1000288. doi: 10.1371/journal.pbio.1000288 (PMC2811158; doi:10.1371/journal.pbio.1000288)
Supplement: Table S1 — Energy distribution between PSI and PSII measured as the fluorescence emission ratio at 730 nm and 685 nm (F730/F685). (0.04 MB DOC) [file pbio.1000288.s002.doc]

| **Table S1.** Energy distribution between PSI and PSII measured as the fluorescence emission ratio at 730 nm and 685 nm (F_730_/F_685_). | | |
| --- | --- | --- |
|  | **F_730_/F_685_** | |
|  | **State 1** | **State 2** |
| WT | 0.99 ± 0.05 | 1.38 ± 0.03 |
| *tap38-1* | 1.49 ± 0.04 | 1.47 ± 0.02 |
| *tap38-2* | 1.32 ± 0.03 | 1.45 ± 0.04 |
| oe*TAP38* | 0.98 ± 0.02 | 1.11 ± 0.03 |
